# Barriers to Renal Transplant in Pakistan

**DOI:** 10.7759/cureus.84850

**Published:** 2025-05-26

**Authors:** Aurangzeb Afzal, Muhammad Ahmad Rauf, Zohra Khanum, Hafiza Sumaira Rahman, Zahid Rafique, Areeba Gulzar, Waqas Rasheed, Jehangir Afzal Mobushar, Beenish Abbas Bajwa

**Affiliations:** 1 Nephrology, Services Hospital Lahore, Lahore, PAK; 2 Epidemiology, Principal Services Institute of Medical Sciences, Lahore, PAK; 3 Medicine, Rashid Hospital, Dubai, ARE; 4 Research, Services Hospital Lahore, Lahore, PAK

**Keywords:** cultural barriers, kidney transplant, patients, punjab human organ transplant authority, socioeconomic status

## Abstract

Background

Kidney transplantation is the preferred treatment for end-stage renal disease (ESRD), offering better survival and quality of life compared to dialysis. However, in Pakistan, multiple socio-economic, legal, and healthcare-related barriers limit the accessibility of kidney transplants. The objective of the current study is to evaluate the barriers to kidney transplant among ESRD patients in Pakistan.

Methods

A cross-sectional study was conducted among 244 ESRD patients undergoing maintenance haemodialysis (MHD) in three major hospitals in Lahore, Pakistan. Data were collected through structured interviews using a comprehensive questionnaire covering socio-demographics, medical history, transplant awareness, donor availability, and legal knowledge.

Results

The mean age of participants was 46 ± 15 years, with 57.8% males and 42.2% females. Awareness of kidney transplantation was low, with only 29.9% expressing interest in undergoing transplantation. Only 5.7% had a legally eligible donor, primarily siblings (28.5%) and spouses (14.2%). Awareness of the Punjab Human Organ Transplant Authority (PHOTA) was poor (15.6%). Legal knowledge gaps were prominent, as only 9.8% knew that unrelated donors were prohibited by law and 4.9% were aware that financial compensation for organ donation is illegal. Economic constraints, lack of awareness, religious misconceptions, and gender disparities were identified as key barriers. Moreover, 3.3% of potential donors initially agreed but later withdrew, and 2.5% were rejected during medical evaluations.

Conclusion

The study highlights significant socioeconomic, cultural, and legal barriers to kidney transplantation in Pakistan. Targeted awareness campaigns, improved healthcare infrastructure, legal reinforcement, and financial assistance programs are needed to bridge the gap between demand and access to kidney transplants. Addressing these barriers can enhance transplantation rates and improve patient outcomes.

## Introduction

Kidney transplantation is acknowledged as the optimal treatment for end-stage renal disease (ESRD) worldwide, offering better survival rates and quality of life compared to dialysis. However, in Pakistan, the implementation of this life-saving procedure encounters numerous obstacles, resulting in a significant gap between the demand for and the availability of kidney transplants.

Pakistan, with a population exceeding 220 million, faces a growing incidence of ESRD, which is attributed to factors such as diabetes, hypertension, and glomerulonephritis. The estimated incidence of ESRD is approximately 100 per million population, translating to thousands of new patients requiring renal replacement therapy every year. Unfortunately, due to limited resources and infrastructure, a substantial proportion of these patients do not receive adequate treatment, leading to high morbidity and mortality rates [1]. Renal transplantation in Pakistan commenced in 1979, primarily utilizing living related donors. Despite advancements in medical technology, the country performs approximately 1,000 kidney transplants annually, a figure insufficient to meet the high demand. Moreover, it is estimated that around 50% of these transplants are conducted illegally, involving unrelated donors, which raises ethical and medical concerns [2].

Barriers to kidney transplantation

A significant portion of Pakistan's population lives below the poverty line, earning less than $1 per day. This economic hardship limits access to both dialysis and transplantation services, as the costs associated with these treatments are prohibitive for many patients [1]. Sociocultural beliefs and a lack of awareness about organ donation impede the development of a robust transplantation program. Misconceptions and religious concerns contribute to the reluctance in both donating and receiving organs [3].

Despite the enactment of the Transplantation of Human Organs and Tissues Act in 2010, which aimed to regulate organ transplantation and curb illegal trade, challenges persist. The illegal organ trade continues to thrive, with reports indicating that half of the renal transplants are performed illicitly, often exploiting vulnerable populations [4]. The healthcare system in Pakistan faces numerous challenges, including inadequate funding, insufficient facilities, and a scarcity of trained medical personnel. These limitations hinder the establishment and maintenance of effective transplantation programs [5]. Gender disparities in organ donation and transplantation are evident, with women often being donors and men predominantly being recipients. This imbalance reflects broader societal norms and inequities that need to be addressed to ensure fair access to transplantation services [6].

Global perspective and potential solutions

The challenges faced by Pakistan are not unique; many developing countries encounter similar obstacles in providing equitable access to kidney transplantation. Strategies such as establishing sustainable transplantation programs, enhancing public awareness, and implementing robust legal frameworks have been suggested to overcome these barriers [7]. Additionally, international collaborations and kidney exchange programs have been proposed to alleviate financial and logistical constraints associated with transplantation [8].

This aim of the current research work focuses on assessing transplant awareness, donor availability, legal knowledge, and patient perceptions through a cross-sectional survey of ESRD patients on maintenance of hemodialysis.

## Materials and methods

Study design and setting

This cross-sectional study was conducted to identify the barriers to kidney transplantation among patients undergoing maintenance hemodialysis (MHD) in Lahore, Pakistan. The study was carried out in three major hospitals of Lahore (Services Hospital, Lahore General Hospital, and Mayo Hospital) to ensure a representative sample of the patient population.

Study population and sampling

The study included adult patients (≥18 years) diagnosed with ESRD and undergoing MHD for at least six months. Patients who had previously undergone kidney transplantation or were on the waiting list for transplantation were also included. A total of 244 participants were recruited using a convenience sampling technique. Patients with severe cognitive impairment or those unwilling to participate were excluded from the study.

Data collection

Data were collected through structured interviews using a validated questionnaire from June 2023 to December 2023. The questionnaire was designed to assess sociodemographic characteristics, medical history, knowledge, and perceptions regarding kidney transplantation, availability of potential donors, and awareness of transplantation laws and regulations.

Sample size calculation procedure

The sample size calculation for this study was conducted using the OpenEpi online software (https://www.openepi.com/). Based on the anticipated proportion, it was found that 81.5% of participants cited the lack of a donor as the main reason for not choosing to undergo a transplant [9]. A 95% confidence interval and a 5% margin of error were used in the estimation. The calculated sample size for this study was recommended to be 244 patients.

Study variables 

The primary variables assessed included the following: 

Sociodemographic factors: Age, gender, education level, employment status, and socioeconomic background.

Medical history: Duration of dialysis, comorbid conditions, previous transplant history, and awareness of transplant eligibility.

Awareness and perceptions: Knowledge about kidney transplantation, legal aspects, donor availability, and religious or cultural beliefs influencing transplantation decisions.

Barriers to transplantation: Economic constraints, lack of donor availability, legal and ethical concerns, and healthcare infrastructure limitations.

Ethical considerations

Ethical approval for the study was obtained from the relevant institutional review board (IRB/2023/1176/SIMS). Informed consent was obtained from all participants before data collection. Confidentiality and anonymity of the participants were maintained throughout the study, and data were stored securely.

Data analysis

The collected data were analyzed using Statistical Product and Service Solutions (SPSS, version 25; IBM SPSS Statistics for Windows, Armonk, NY). Descriptive statistics, such as frequencies and percentages, were used to summarize categorical variables, while means and standard deviations were calculated for continuous variables.

## Results

Sociodemographic and health history of the study population

The study population consisted of 244 individuals, with a mean age of 46 ± 15 years. The majority of participants (68.9%) were over 40 years old, while 31.1% were younger than 40. Males comprised 57.8% of the study population, while females accounted for 42.2%.

Regarding MHD, 47.5% of participants underwent dialysis twice weekly, whereas 52.5% were on a thrice-weekly regimen. The distribution of blood groups among the participants showed that the most common group was B+ (29.5%), followed by A+ (23.8%) and O+ (14.8%), with a smaller percentage having AB+ (11.1%) and B- (3.3%). Additionally, 17.6% of participants were unaware of their blood group. Screening for hepatitis B, hepatitis C, and HIV revealed that 40.6% had undergone PCR testing, while 56.6% had not. Among those diagnosed, 38.1% had received treatment, whereas 58.6% had not. A small proportion (2.9%-3.3%) preferred not to disclose their status.

Regarding past medical history, 2% had experienced a previous transplant and graft loss, 0.4% had a history of malignancy, and an equal percentage reported psychosis or substance abuse. Smoking was reported by 3.3% of participants, while 3.3% also had an ongoing infection (Table 1).

**Table 1:** Sociodemographic and health history of the study population

Variables	Categories	N (%)
Age	46 ± 15 years	
Age Groups (Years)	< 40	76 (31.1)
	> 40	168 (68.9)
Gender	Male	141 (57.8)
	Female	103 (42.2)
On Maintenance Hemodialysis (MHD)	Twice weekly	116 (47.5)
	Thrice weekly	128 (52.5)
Blood Group	A+	58 (23.8)
	AB+	27 (11.1)
	B-	8 (3.3)
	B+	72 (29.5)
	O+	36 (14.8)
	Don’t know	43 (17.6)
If Hep B, C, HIV (+ve) PCR Done?	Yes	99 (40.6)
	No	138 (56.6)
	Prefer not to answer	7 (2.9)
If Hep B, C, HIV (+ve) Treatment Taken	Yes	93 (38.1)
	No	143 (58.6)
	Prefer not to answer	8 (3.3)
H/O Previous Transplant (Reason for Loss of Graft)	Yes	5 (2.0)
	No	232 (95.1)
	Prefer not to answer	7 (2.9)
H/O Malignancy	Yes	1 (0.4)
	No	236 (96.7)
	Prefer not to answer	7 (2.9)
H/O Psychosis	Yes	1 (0.4)
	No	236 (96.7)
	Prefer not to answer	7 (2.9)
H/O Substance Abuse	Yes	1 (0.4)
	No	235 (96.3)
	Prefer not to answer	8 (3.3)
H/O Smoking	Yes	8 (3.3)
	No	227 (93.0)
	Prefer not to answer	9 (3.7)
Any Ongoing Infection	Yes	8 (3.3)
	No	229 (93.9)
	Prefer not to answer	7 (2.9)

Cognizance of renal transplantation

Interest in kidney transplantation was noted in 29.9% of participants, whereas 65.6% were not interested, and 4.5% were unsure. Awareness of the Punjab Human Organ Transplant Authority (PHOTA) was low, with only 15.6% reporting knowledge of the organization, while 74.2% were unaware. Regarding potential live-related donors, 5.7% had an eligible donor, whereas 77.5% did not, and 16.8% were uncertain. Among the 14 individuals with a donor, the most common donor relationships included siblings (28.5%), husbands (14.2%), and sons (14.2%). Among potential donors, 21.4% had a known disease, 57.1% were disease-free, and 21.4% were unsure. When asked about financial benefits in donation, 14.2% believed it was involved, while 71.2% denied such involvement (Table 2).

**Table 2:** Cognizance of renal transplantation among the study population

Questions	Responses	N (%)
Are you interested in a kidney transplant?	Yes	73 (29.9)
	No	160 (65.6)
	Not sure	11 (4.5)
Do you know about the HOTA or Punjab Human Organ Transplant Authority (PHOTA)?	Yes	38 (15.6)
	No	181 (74.2)
	Prefer not to answer	25 (10.2)
Do you have a live, related potential donor according to the Law (PHOTA)?	Yes	14 (5.7)
	No	189 (77.5)
	Not sure	41 (16.8)
What’s the relation of the donor to you? (N=14)	Husband	2 (14.2)
	Son	2 (14.2)
	Brother	1 (7.1)
	Sister	3 (21.4)
	Others	6 (42.8)
Does he/she have a known disease? (N=14)	Yes	3 (21.4)
	No	8 (57.1)
	Not sure	3 (21.4)
Do you think there is a financial benefit involved for the donor?	Yes	2 (14.2)
	No	10 (71.2)
	Not sure	2 (14.2)

Knowledge of kidney transplant laws and regulations

**Figure 1:**
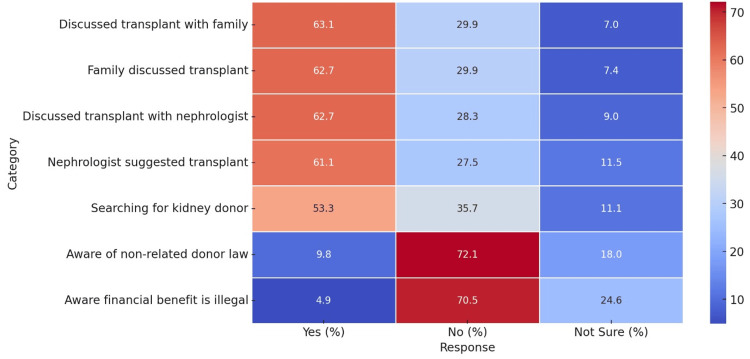
Knowledge of kidney transplant laws and regulations

Discussion of kidney transplantation within families was reported by 63.1% of participants, while 29.9% had not engaged in such discussions. Similarly, 62.7% had received input from family members regarding transplantation. Consultation with nephrologists revealed that 62.7% had discussed transplantation with their physician, and 61.1% had been advised about it. More than half (53.3%) were actively searching for a donor, while 35.7% were not. Knowledge regarding legal restrictions on kidney donation was limited, as only 9.8% were aware that non-related persons could not donate a kidney. Additionally, 4.9% knew that financial compensation for donation is illegal, whereas 70.5% were unaware, and 24.6% were unsure (Figure 1).

Preoperative evaluation for renal transplantation

Among potential donors, 3.3% had initially agreed but later refused to donate, and 2.5% were rejected during the transplant workup. Only 3.7% of participants reported having a willing donor undergoing a pre-transplant evaluation. Fitness for kidney transplantation was assessed, revealing that 1.2% had been deemed unfit by their nephrologist, while 72.5% were considered suitable candidates. Religious concerns were minimal, as only 0.4% cited religious reasons for not opting for transplantation (Table 3).

**Table 3:** Preoperative evaluation for renal transplantation

Questions	Responses	N (%)
Did any of potential donors refuse after agreeing initially?	Yes	8 (3.3)
	No	174 (71.3)
	Not sure	62 (25.4)
Did a donor get rejected during workup for kidney transplant?	Yes	6 (2.5)
	No	175 (71.7)
	Not sure	63 (25.8)
Is there a potential donor who is willing, and pre-transplant workup is underway?	Yes	9 (3.7)
	No	183 (75.0)
	Not sure	52 (21.3)
Have your nephrologist labelled you not fit for kidney transplant?	Yes	3 (1.2)
	No	177 (72.5)
	Not sure	64 (26.2)
Is there any religious reason for not opting transplant?	Yes	1 (0.4)
	No	177 (72.5)
	Not sure	66 (27.0)

Perception and awareness of organ transplants

Only 13.1% of participants believed in volunteering for organ donation after death, while 48.8% opposed it, and 38.1% were unsure. Regarding the underlying causes of ESRD, 1.6% had dysfunctional or obstructed urinary bladders, and 1.2% had kidney stones as the primary cause. The majority (55.7%) were unaware of the specific disease leading to their ESRD. Active disease status was discussed with nephrologists, and 3.3% were advised to wait for transplantation due to ongoing disease activity. Only 0.8% were informed that they would never qualify for a transplant, whereas 89.3% were not given such a restriction (Table 4).

**Table 4:** Perception and awareness of organ transplants

Questions	Responses	N (%)
Do you think we should volunteer to donate kidney after we die?	Yes	32 (13.1)
	No	119 (48.8)
	Not sure	93 (38.1)
Have you been diagnosed of dysfunctional/obstructed urinary bladder as cause of ESRD?	Yes	4 (1.6)
	No	194 (79.5)
	Not sure	46 (18.9)
Do you have kidney stones as the cause of ESRD?	Yes	3 (1.2)
	No	200 (82.0)
	Not sure	41 (16.8)
Do you know the primary disease leading to ESRD?	Yes	57 (23.4)
	No	136 (55.7)
	Not sure	51 (20.9)
Did your nephrologist say that the disease leading to kidney failure is still Active and you should wait?	Yes	8 (3.3)
	No	182 (74.6)
	Not sure	54 (22.1)
Have doctor said that you can never be transplanted?	Yes	2 (0.8)
	No	218 (89.3)
	Not sure	24 (9.8)

## Discussion

The present study offers a comprehensive analysis of the sociodemographic characteristics, health history, and awareness levels concerning renal transplantation among patients undergoing MHD in Lahore, Pakistan. Understanding these factors is crucial for improving patient outcomes and addressing the challenges associated with ESRD management in the region.

The study population had a mean age of 46 ± 15 years, with a predominance of individuals over 40 years old (68.9%). This age distribution aligns with global trends, where the incidence of ESRD increases with age due to cumulative exposure to risk factors such as hypertension and diabetes mellitus [10]. The male-to-female ratio in our cohort was 1.37:1, indicating a higher prevalence of ESRD among males. Similar gender disparities have been reported in other studies, suggesting potential biological and behavioral factors contributing to this difference [11].

Approximately half of the participants were on a twice-weekly dialysis regimen (47.5%), while the remainder received dialysis thrice weekly (52.5%). The choice between these regimens often depends on factors such as residual renal function, patient comorbidities, and resource availability [12]. The distribution of blood groups revealed B+ as the most common (29.5%), followed by A+ (23.8%) and O+ (14.8%). This distribution is consistent with the general Pakistani population, where B+ is prevalent [13].

Notably, 40.6% of participants had undergone PCR testing for hepatitis B, hepatitis C, and HIV, with 38.1% of diagnosed individuals receiving treatment. These findings highlight a gap in screening and treatment, which is concerning given the high risk of viral infections in dialysis patients [14]. Regular screening and timely treatment are essential to prevent complications and improve patient survival.

The prevalence of previous transplants was low (2%), and histories of malignancy, psychosis, or substance abuse were rare (0.4% each). Smoking was reported by 3.3% of participants, aligning with global trends where smoking rates are lower among dialysis patients due to health counselling [15]. Ongoing infections were present in 3.3% of the cohort, underscoring the need for vigilant infection control measures in dialysis units.

A significant finding was that only 29.9% of participants expressed interest in kidney transplantation, while 65.6% were not interested, and 4.5% were unsure. This low interest may stem from limited awareness, cultural beliefs, or misconceptions about transplantation [3]. Furthermore, awareness of the PHOTA was notably low, with only 15.6% of participants familiar with the organization. This lack of awareness may contribute to misconceptions about the legality and safety of transplantation procedures [16].

Only 5.7% of participants reported having a live-related potential donor, with siblings being the most common donors (28.5%). The scarcity of potential donors is a significant barrier to transplantation, often leading patients to remain on long-term dialysis [17]. Addressing this issue requires public awareness campaigns to encourage organ donation and dispel myths surrounding the process.

The study revealed limited knowledge regarding legal restrictions on kidney donation, with only 9.8% aware that non-related persons cannot donate kidneys according to the law, and a mere 4.9% knowing that financial compensation for donation is illegal. This lack of awareness may perpetuate illegal organ trade and exploitation [4]. Educational initiatives targeting both patients and the general public are essential to promote ethical transplantation practices and protect vulnerable populations.

Encouragingly, 63.1% of participants had discussed kidney transplantation with their families, and 62.7% had consulted their nephrologists about the option. Such discussions are crucial for informed decision-making and aligning treatment plans with patient preferences [18]. However, the fact that only 53.3% were actively searching for a donor indicates potential barriers, such as fear, misinformation, or logistical challenges, hindering the pursuit of transplantation.

A small proportion of potential donors (3.3%) initially agreed but later refused to donate, and 2.5% were rejected during the transplant workup. These findings highlight the complexities involved in donor selection and the importance of thorough medical and psychological evaluations [19]. Notably, 72.5% of participants were considered suitable candidates for transplantation by their nephrologists, suggesting that a majority could benefit from the procedure if other barriers are addressed.

Only 13.1% of participants believed in volunteering for organ donation after death, while 48.8% opposed it, and 38.1% were unsure. This reluctance may be rooted in cultural, religious, or educational factors [3]. Engaging religious and community leaders in educational campaigns could help shift perceptions and increase acceptance of posthumous organ donation.

One of the important barriers to kidney transplant is the lack of deceased donor programs. In many of the major countries where deceased kidney transplants are functional, it is considered a major contribution towards kidney transplants. In Pakistan, the process of kidney transplant depends only on living donors. In this regard, educating the public and the formation of legislation could help in fulfilling this gap [20].

Furthermore, due to geographical differences between rural and urban areas, most of the kidney transplant centres are located in major cities of Pakistan, such as Lahore and Karachi. Lack of information, lack of transportation, travelling costs, and distance create hurdles for many ESRD patients. Another factor that contributes major factor for ESRD patients towards kidney transplant is depression, anxiety, and psycho-social barriers [21].

Finally, financial burden is also supposed to be a main concern. As kidney transplant is much feasible and cost-effective as compared to the costly and painful kidney dialysis procedure.

Limitations

While this study provides valuable insights, it is essential to acknowledge its limitations. The cross-sectional design captures a single point in time, limiting the ability to establish causality. Additionally, self-reported data may be subject to recall bias. Future longitudinal studies are needed to explore how awareness and attitudes toward transplantation evolve over time.

## Conclusions

This study highlights critical gaps in awareness, interest, and legal knowledge regarding renal transplantation among MHD patients. Targeted educational campaigns and policy interventions are necessary to enhance awareness, facilitate donor availability, and promote ethical transplant practices in Pakistan. Strengthening physician-patient communication and integrating community-driven initiatives may significantly improve transplantation rates and patient outcomes.
